# An exploratory analysis of demography and movement patterns of dogs: New insights in the ecology of endemic Rocky Mountain-Spotted Fever in Mexicali, Mexico

**DOI:** 10.1371/journal.pone.0233567

**Published:** 2020-05-21

**Authors:** Andrés M. López-Pérez, Libertad Orozco, Oscar E. Zazueta, Maria Fierro, Paola Gomez, Janet Foley

**Affiliations:** 1 School of Veterinary Medicine, University of California, Davis, California, United States of America; 2 Área de Investigación en Biología de la Conservación, Departamento de Ciencias Ambientales, Universidad Autónoma Metropolitana-Lerma, Lerma de Villada, Estado de Mexico, Mexico; 3 Secretaria de Salud Pública del Estado de Baja California, Mexicali, Mexico; 4 Public Health Department, Imperial County, El Centro, California, United States of America; Kansas State University, UNITED STATES

## Abstract

An outbreak of Rocky Mountain spotted fever (RMSF) transmitted by the brown dog tick (*Rhipicephalus sanguineus* sensu lato) has emerged as a major human and animal health concern in Mexicali, Mexico. Due to high rates of brown dog tick infestation, susceptibility, and association with humans, dogs serve as sentinels and have a key role in the ecology of RMSF. A cross-sectional household questionnaire study was conducted in six rural and urban locations to characterize dog ecology and demography in RMSF high-and low-risk areas of Mexicali. In addition, we tracked movement patterns of 16 dogs using a GPS data logger. Of 253 households, 73% owned dogs, and dog ownership tended to be higher in high-risk areas, with a mean dog:human ratio of 0.43, compared with 0.3 in low-risk areas. Dogs in high-risk areas had higher fecundity and roamed more, but the dog density and numbers of free-roaming dogs were comparable. There was a higher proportion of younger dogs and lower proportion of older dogs in high-risk areas. The high proportion of immunologically naïve puppies in high risk areas could result in a lack of herd immunity leading to a more vulnerable dog and human population. The marked increase of space use of free-roaming dogs in high-risk areas suggests that unrestrained dogs could play an important role in spreading ticks and pathogens. As means to limit RMSF risk, practical changes could include increased efforts for spay-neuter and policies encouraging dog restraint to limit canine roaming and spread of ticks across communities; due to dog density is less impactful such policies may be more useful than restrictions on the number of owned dogs.

## Introduction

Domestic dogs (*Canis lupus familiaris*) have featured in human civilization for tens of thousands of years, providing protection, assistance hunting, companionship, and other services [1, 2]. However, dogs share pathogens and parasites with humans, in some cases bridging disease from other animals including wildlife to people, serving as reservoirs for shared disease agents, and acting as sentinels for human risk [3]. Dogs with greater roles in human disease ecology include those that interact with wildlife or roam free, those in populations characterized by rapid turnover (due to high mortality or fecundity rates), and those subject to low standards of veterinary care including vaccination. Examples of diseases associated with pet dogs include rabies, which is endemic in some areas where dogs are not restrained or vaccinated [4], and the tapeworm *Echinococcus* for which dogs and other canids can be important sources of environmental contamination [5].

Rocky Mountain spotted fever (RMSF), a potentially fatal disease of dogs and people caused by the bacterium *Rickettsia rickettsii*, is the most important rickettsiosis in North America [6, 7]. Tick vectors of RMSF include the American dog tick (*Dermacentor variabilis*), the wood tick (*D*. *andersoni*), the brown dog tick (*Rhipicephalus sanguineus sensu lato*), as well as several *Amblyomma* tick species (e.g. *A*. *cajennense*, *A*. *imitator*, and *A*. *parvum*) [8–12]. While *Dermacentor* spp. ticks are essentially sylvatic, preferring wild small mammal hosts in immature stages, all feeding stages of the brown dog tick prefer dogs, such that this tick is unusual in being fully peri-domestic. The abundance of the brown dog tick is determined by the number of dogs, especially stray dogs, in a community [8, 13, 14], with ticks sometimes spilling over to feed on humans. Rickettsial infections in dogs influence prevalence in ticks and serve to amplify the presence of the pathogen [8, 15].

Epidemics of RMSF have been associated with high numbers of stray dogs and uncontrolled tick infestations in eastern Arizona in the United States and in the Mexican states of Sonora and Baja California [8, 13]. At least 250 cases with 19 deaths occurred among American Indians in Arizona from 2003–2012, prompting an aggressive and successful response by public health officials incorporating long-acting acaricidal collars on dogs, treatment of the environment, and spay-neuter plus tethering of dogs [13]. The RMSF epidemic in Baja California is much larger than those in Arizona. Since 2008, an outbreak of RMSF is ongoing in Mexicali, a city of 700,000 people immediately south of the border with the US, affecting at least 1000 people and countless dogs, with a high human mortality rate of approximately 40% [16]. In Mexico, the vector is a distinct tropical lineage of brown dog tick, in contrast with epidemics in Arizona associated with the temperate lineage [17]; increased warming and drying associated with climate change may permit the tropical lineage ticks to expand northward, with evidence that canine exposure is increased directly north of the US/Mexico border [18].

Epidemiological determinants of RMSF in Mexico are not well-understood. Ticks can acquire the infection from infected dogs as well as transovarially through passage of the bacterium through tick eggs [19, 20]. However, surveillance may reveal very low prevalence of antibodies in dogs and low proportion of *Rickettsia*-positive ticks [21], possibly due in part to fine spatial scale endemic foci as well as local disease extinction and introduction of disease to new areas. Across Mexicali, there appears to be a higher risk for human cases and seropositive dogs in some neighborhoods on the periphery of the city and in agricultural small towns (ejidos) in the valley that extends to the southeast of the city [21–23]. Even though local elevations in tick numbers increase risk, there have been cases of RMSF and documented brown dog tick infestations even in homes without dogs [21]. Although RMSF may be fatal in dogs, animals that survive infection may develop immunity for some period of time, suggesting that abundant puppies could reduce herd immunity and enhance the persistence of the pathogen in a community. Human decisions about dog ownership including allowing animals to roam, not treating the dogs with acaricides, and keeping dogs outside could all impact tick infestation risk as well as risk of RMSF both in the dog and in humans in the household.

Our goals in this study were to document dog population size, dog:human ratios, and demographic characteristics of canine populations in neighborhoods characterized as high and low-risk for RMSF, and to evaluate canine movement patterns in these differing areas.

## Materials and methods

### Study area

Work was approved by the University of California Davis Institutional Animal Care and Use Committee (protocol # 20483) and the Hospital General de Mexicali Research Committee protocol # 02-01-HGMXL/ISESALUD/CDC/UCDAVIS-2019-08-07-250.

**Fig 1:**
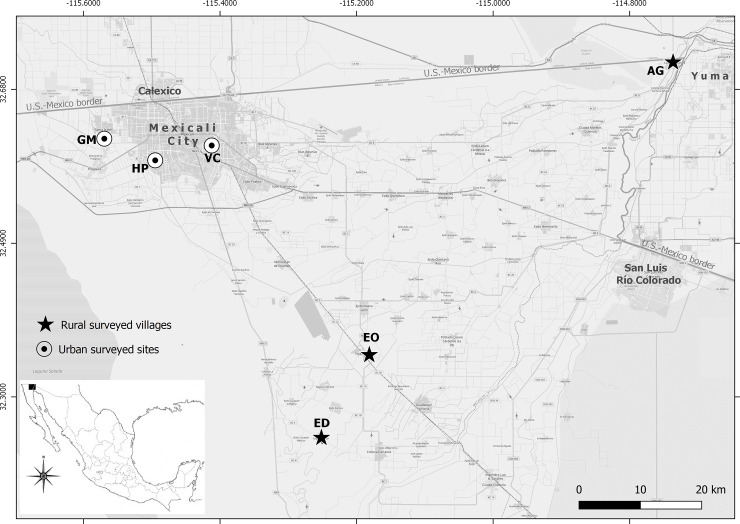
Sampling sites of rural and urban areas in Mexicali Valley, Mexico. Capital letters refer to locations (Urban neighborhoods: GM: Gabriela Mistral, HP Hacienda Los Portales, VC: Venustiano Carranza; rural areas: ED: Ejido Durango, EO: Ejido Oaxaca, AG: Algodones). Reprinted from the OpenStreetMap vector basemap hosted by Environmental Systems Research Institute (Esri) and provided under a CC BY 4.0 license, (Map data © OpenStreetMap contributors, Map layer by Esri 2019).

The study was conducted during August 2019 (when ticks have increased abundance and activity [24]) at six locations, including three rural villages and three urban areas in and near Mexicali in northwestern Mexico (Fig 1). Mexicali is located within the lower Colorado River Delta in the Sonoran Desert, and comprises a mosaic of landscapes dominated by urban and rural settlements and agriculture, with some areas of riparian and shrub-dominated vegetation. The climate is arid with a mean annual precipitation of 55.4 mm and highly variable temperatures from 6°C—42°C with a mean annual temperature of 22.4 °C.

The locations chosen for study included two urban and two rural sites that were categorized as high-risk for RMSF (Gabriela Mistral, 32°37'N, 115°34'W, and Hacienda Portales, 32°35'N, 115°29'W in Mexicali City and Ejido Durango, 32°14'N, 115°15'W and Algodones, 32°42'N, 114°44'W in the agricultural valley), and one low-risk rural and urban control (Venustiano Carranza, 32°36'N, 115°24'W in Mexicali City and Ejido Oaxaca, 32°21'N, 115°10'W in the valley). RMSF risk was classified based on a previous study [21], defining a high-risk area as the one that meet three criteria 1) at least one confirmed *R*. *rickettsii*-positive tick; 2) a prevalence of RMSF antibodies in dogs > 30% (cut-off titer >1:64); and 3) a confirmed human case of RMSF within 12 months preceding the study.

### Household questionnaires

A cross-sectional household questionnaire was conducted. We randomly selected three blocks from each rural town and urban neighborhood. At each block, questionnaires were deployed by choosing a random house and asking if the inhabitants consented to participate. If consent was not provided or the household did not own a dog (s), the team continued around the block and the surrounding areas until obtaining consent of at least 10 dog-owning households. Questionnaires were delivered orally in Spanish by students from the Medical School of Baja California Autonomous University and personnel of the Baja California Secretary of Health. Questions focused on number of dogs, demography of dogs, female dog reproductive history, and household dog ownership (S1 Table). At each sampled household, the person at home and best able to answer questions related to dogs and their care was asked for a verbal consent to participate in the survey. Institutional review board (IRB) approval was not required as all questions specifically related to care and husbandry of the dogs and were covered under the veterinarian-client relationship.

Households were classified as dog-owning (DOHH) and non-dog owning (NDOHH). For DOHH, we recorded the number of dogs in the household, and we asked the owners questions relating to reasons for owning the dog(s) (guardian, shepherd, pet, hunting), origin of dogs (acquired from neighbor or family, bought, found or adopted, or born at home), number of sterilized animals, the presence of free-roaming dogs of unknown origin and if each animal was allowed to roam freely. We also asked how many dogs were female and how many litters had been produced by female dogs. The area of each neighborhood and rural village was determined by drawing a perimeter of the polygon using QGIS 3.4 (QGIS Development Team, 2009).

### GPS-tracking data collection

Sixteen free-roaming male dogs were collared and monitored from August 10–22, 2019: these included five dogs (three were 1-yr old, one was 4 yr-old, and one was 5-yr old) from a high-risk rural village (Ejido Durango), five (ages 1-yr for two dogs and one dog each aged 2, 3, and 4-yr old) from low-risk rural Ejido Oaxaca, three (two 2-yr old and one 6-yr old) from high-risk urban Colonia Hacienda Portales and three (ages 1, 2, and 5-yr old) from low-risk urban Colonia Venustiano Carranza. None of these dogs was neutered. All collaring and tracking was done after obtaining written informed consent from the owners. Collars were lightweight nylon with an attached motion-detecting GPS-data logger (Igot-U GT600, Mobile Action Technology, Taipei, Taiwan) configurated to record a location every 10 min. Logger data were downloaded into the i-gotU Sport Analyzer software (Mobile Action Technology). Each track was visually inspected for anomalies and gaps in data collection, and GPS coordinates for each recorded location were extracted into a spreadsheet. Locations were visualized and analyzed with QGIS. We estimated the area occupied (AO) by each dog by calculating the minimum convex polygon (MCP) with the Minimum Bounding Geometry tool. We determined the number of houses that each dog visited (NHV) as the number of houses within each occupied area. Maximum distance moved (MDM) by each dog was calculated as the maximum distance among location points using the Measure tool.

### Statistical analysis

Summary statistics were calculated including mean number of dogs per DOHH for each neighborhood. We estimated the dog population size and dog density for each site using the data derived from the questionnaires (proportion of households that own dogs and mean numbers of dogs per household). The dog population size was calculated by multiplying the number of inhabited households by the proportion of dog owning household and then by the mean number of dogs per household that owned at least one dog in each neighborhood and rural village. The dog population density was calculated by dividing the estimated dog population by the area of each site. The dog:human ratio was estimated by dividing the total human population by the total number of dogs calculated for each location. Human population size and the number of inhabited households were taken from the human census of 2016 performed by the Instituto Nacional de Estadística y Geografia of Mexico [25]. Female reproduction information was used to calculate mean litter size and female fecundity. Fecundity was determined as the number of female offspring per female per year *m(x)*, i.e. proportion of breeding females (>12months) and the mean number of pups/female in the past 12 months following [26]. This fecundity estimation assumes a 1:1 male:female ratio at birth.

Associations between RMSF risk level and numbers of free roaming dogs, dog function, and dog origin were assessed using chi-square tests for rural and urban areas. Fecundity and dog density were compared by risk level (high vs low), and landscape (rural vs urban), using a two-sided Student’s t-test with equal variances. Two-way ANOVA models were performed to evaluate for effects of risk level (high vs low) and landscape (rural vs urban) on means AO, MDM, and NHV of dogs surveyed. Data were analyzed with the statistical program “R” (R-Development Core Team, 2015) with P<0.05 used as a cutoff to infer statistical significance.

## Results

### Household dog ownership patterns

The mean proportions of households sampled in high and low-risk areas were 5.9% and 8.1%, respectively. Of 253 households interviewed in this study, 182 (73%) were DOHH with a total of 346 dogs. The percentage of DOHH ranged from 63.3 to 85.7% in rural and urban areas and tended to be higher in RMSF high-risk (76.1%) vs. low-risk areas (64.9%, X^2^ = 3.68, df = 1, p = 0.055, Table 1). Overall, there was an average of 1.9 ± 0.05 dogs per DOHH. Mean dog numbers did not differ significantly between RMSF high (1.9 ± 0.1 dogs) and low-risk areas (1.8 ± 0.2 dogs; t = 0.371, df = 106, p = 0.712), or between urban (1.8 ± 0.1 dogs) and rural areas (2.0 ± 0.1 dogs; t = 0.981, df = 180, p = 0.328). Free roaming dogs of unknown ownership were reported in all locations, ranging from 36.6% to 93.3% of dogs present. A higher percentage was reported in high-risk (76.6–93.3%) compared with low-risk (36.6%) in rural areas (X^2^ = 21.8, df = 1, p-value = 0.000), but did not differ between high (83.3–87.4%) and low-risk (90.3%) neighborhoods in urban areas (X^2^ = 0.47, df = 1, p-value = 0.494) (Table 1).

**Table 1:** Dog ownership patterns, population size and densities in RMSF risk-urban and rural areas in and near Mexicali, Mexico.

Landscape	Rural		Urban			
Locations	Ejido Oaxaca	Ejido Durango	Algodones	Venustiano Carranza	Gabriela Mistral	Hacienda Portales
RMSF risk level^1^	Low	High	High	Low	High	High
Households interviewed	45	35	46	49	36	42
DOHH %^2^	66.7	85.7	65.2	63.3	83.3	73.8
Free-roaming dogs of unknown ownership %^3^	36.6	76.6	93.3	90.3	83.3	87.4
No. of owned dogs in survey	56	61	62	57	58	52
Mean dogs per household	1.9	2.0	2.1	1.8	1.9	1.7
No. of houses in neighborhood	727	439	1528	491	367	1528
Estimated number of households with dogs in neighborhood	485	376	997	311	306	1128
Estimated dog population size per neighborhood	905	765	2059	571	591	1892
Human population size per neighborhood	2677	1593	5398	1804	1415	5272
Dog:human ratio	0.3	0.5	0.4	0.3	0.4	0.4
Location (neighborhood) area (km^2^)	2.1	1.6	2.4	0.3	0.7	0.8
Density (Dogs/km^2^)	426.7	479.4	843.8	1729.3	812.9	2260.2

^1^High-risk level criteria: 1) at least one confirmed *R*. *rickettsii*-positive tick; 2) a prevalence of RMSF antibodies in dogs > 30%; and 3) a confirmed human case of RMSF within 12 months preceding the study

^2^ DOHH = dog-owning household

^3^ Estimated from data at the household level.

Rural areas had a higher percentage of free-roaming dogs than urban areas (62.9% vs 49.1%; Table 2). There was also a slightly lower percentage of dogs allowed to freely roam in high compared with low-risk settlements in rural areas (54.5 vs 59.3%), but these differences were not statistically significant (Table 2).

**Table 2:** Chi-square tests for dog ownership practices comparing frequencies of the free-roaming activity, dog function and source of dogs among dogs from rural and urban areas with two levels of RMSF risk in and near Mexicali, Mexico.

	RMSF risk level^1^					Landscape				
	High n (%)	Low n (%)	X^2^	df	*P*-value	Rural n (%)	Urban n (%)	X^2^	df	*P*-value
**Free-roaming activity**			0.71	1	0.400			6.36	1	**0.012**
Yes	127 (54.5)	67 (59.3)				112 (62.6)	82 (49.1)			
**Dog function**			1.35	1	0.244			0.13	1	0.712
Guard	100 (42.9)	56 (49.6)				79 (44.1)	77 (46.1)			
Pet	133 (57.1)	57 (50.4)				100 (55.9)	90 (53.9)			
**Source of dogs**			4.58	1	0.205			3.68	1	0.298
Gift	118 (50.6)	68 (60.2)				100 (55.9)	86 (51.5)			
Bought	20 (8.6)	12 (10.6)				18 (10.1)	14 (8.4)			
Found	62 (26.6)	20 (17.7)				35 (19.5)	47 (28.1)			
Born	33 (14.2)	13 (11.5)				26 (14.5)	20 (12.0)			

^1^High-risk level criteria: 1) at least one confirmed *R*. *rickettsii*-positive tick; 2) a prevalence of RMSF antibodies in dogs > 30%; and 3) a confirmed human case of RMSF within 12 months preceding the study; Significant p-values are indicated in bold.

Most dogs were considered pets or guard dogs, and none were used as herding or hunting dogs (Table 2). The proportion of guarding and pet dogs did not differ between high and low-risk areas nor within rural and urban areas. The most common source of owned dogs in every neighborhood and rural village was dogs obtained as a gift from a family member or neighbor (from 26% to 40%); the second-most common source was the dog being found (from 8 to 22%, Table 2).

We estimated a population size ranging from 765 to 2059 owned dogs for rural areas and from 571 to 1892 for urban neighborhoods (Table 1). Hacienda Los Portales had the highest dog density with 2260 dogs/km^2^, followed by Venustiano Carranza with 1729 dogs/km^2^. Mean dog density was significantly higher in urban vs. rural areas, but did not differ significantly between RMSF high-risk and low-risk areas (Table 3).

**Table 3:** Statistics summary for dog density and reproductive performance among dogs from rural and urban areas with two levels of RMSF risk in and near Mexicali, Mexico.

Source of Variation	Level^1^	Mean ± SE	d.f.	T	P
**Density** (dogs/km^2^)					
Risk level			4	1.14	0.841
	High	1328.2 ± 406.7			
	Low	619.8 + 193.1			
Landscape			4	-2.30	**0.042**
	Rural	583.3 ± 131.2			
	Urban	1600.8 ± 422.7			
**Reproduction performance**					
*Fecundity*					
Risk level			4	3.30	**0.015**
	High	2.1 ± 0.2			
	Low	1.2 ± 0.1			
Landscape			4	-0.40	0.355
	Rural	1.7 ± 0.3			
	Urban	1.9 ± 0.4			
*Mean litters/female*					
Risk level			4	2.04	0.055†
	High	1.9 ± 0.1			
	Low	1.3 ± 0.4			
Landscape			4	1.22	0.144
	Rural	1.9 ± 0.2			
	Urban	1.5 ± 0.3			

^1^High-risk level criteria: 1) at least one confirmed *R*. *rickettsii*-positive tick; 2) a prevalence of RMSF antibodies in dogs > 30%; and 3) a confirmed human case of RMSF within 12 months preceding the study; Significant p-values are indicated in bold; †Marginally significant.

**Fig 2:**
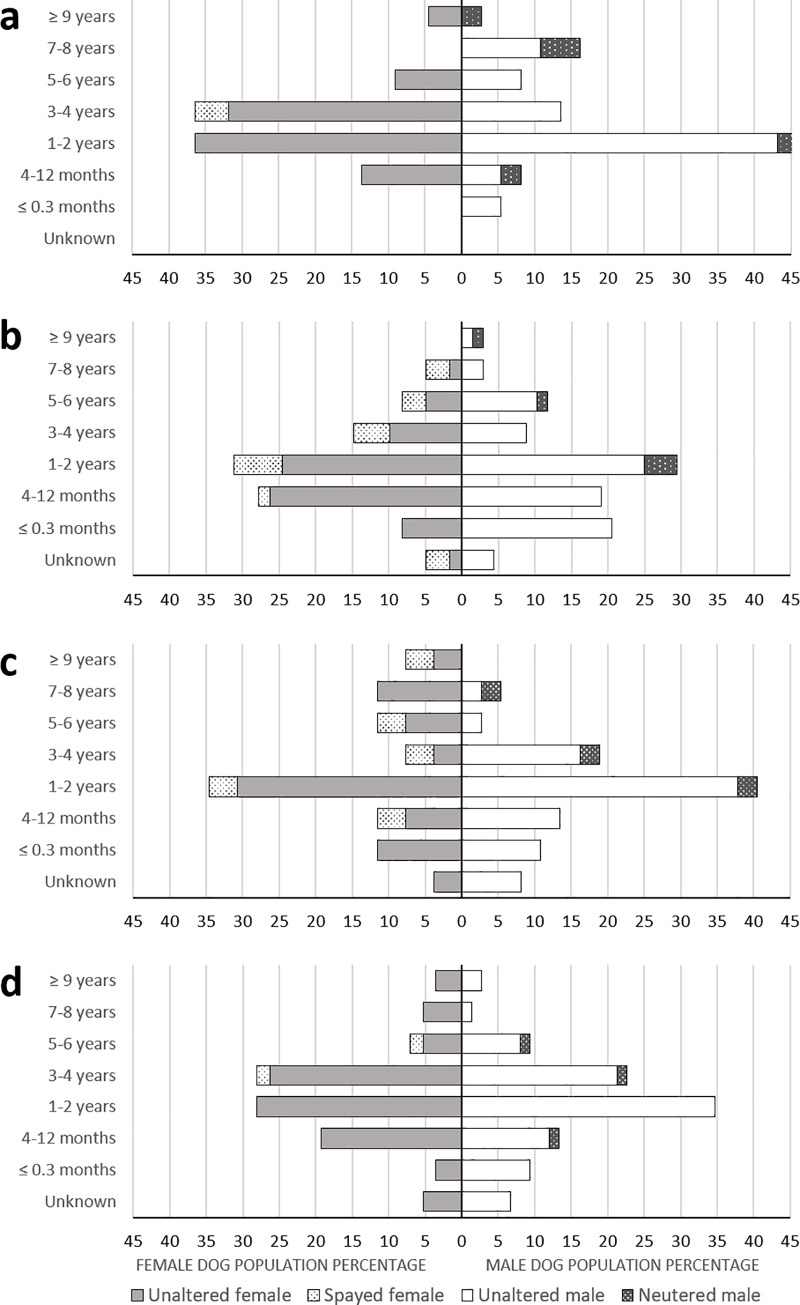
Age-sex population pyramid of owned dogs inhabiting urban RMSF low-risk (a), urban high-risk (b), rural low-risk (c), and rural high-risk (d) areas in and near Mexicali, Mexico.

Among the 383 owned dogs surveyed, 56.7% were male, resulting in a male:female ratio of 1.3:1. However, there were slightly fewer breeding males per female in high-risk (1.3:1) than low-risk areas (1.6:1). The age and sex structure of the owned dog population is compared by landscape and RMSF risk level (Fig 2). Fifty percent of the dog population was between 4 months and 2 years old. However, the percentage of puppies (< 1 yr old) was significantly higher for RMSF high than low-risk areas (30.8% vs 18.9%, X^2^ = 6.40, df = 1, p = 0.011), while the percentage of dogs older than 6 years was significantly lower for RMFS high than low-risk areas (5.7% vs 12.3%, X^2^ = 4.94, df = 1, p = 0.026). Only a small proportion of the dog population was sterilized (9.9%; 38/283), including a slightly higher percentage in female (n = 22; 13.2%) vs male (n = 16; 7.4%) dogs. The proportion of sterilized dogs ranged from 3.5% to 23.0% across sites, with a higher rate in urban than rural (X^2^ = 4.71, df = 1, p-value = 0.03), but did not differ significantly by RMSF risk level (X^2^ = 0.48, df = 1, p = 0.487), and sex (X^2^ = 3.64, df = 1, p = 0.056).

Of 170 female dogs surveyed in this study, there were 122 mature breeding females, of which 86 (70.5%) had already had at least one litter. None of the 48 female dogs less than 12 months old had had a litter, and the longest reproductive lifespan reported was 12 years old. Mean litter size was 3.5 ± 0.4 pups, while mean fecundity rate was 1.8 ± 0.2 female offspring per female per year (Table 4). Fecundity was significantly higher in RMSF high-risk vs. low-risk areas (Table 3); however, no significant differences were detected between rural and urban areas. The overall mean number of litters per female was marginally higher in high-risk areas compared to low-risk areas (Table 3).

**Table 4:** Fecundity *m(x)* and reproduction performance of female dogs from rural and urban areas with two levels of RMSF risk in and near Mexicali, Mexico.

Locations	RMSF risk level^1^	Total number of owned breeding females in survey	Number of litters	Number of puppies last year	Mean number of litters per breeding female	Mean offspring born last year per female	*m(x)*
Rural villages							
Ejido Oaxaca	Low	19	32	43	1.7	2.3	1.1
Ejido Durango	High	23	52	94	2.3	4.1	2.0
Algodones	High	19	35	71	1.8	3.7	1.9
Urban areas							
Venustiano Carranza	Low	19	18	48	0.9	2.5	1.3
Hacienda Portales	High	14	23	50	1.6	3.6	1.8
Gabriela Mistral	High	18	35	92	1.9	5.1	2.6

^1^High-risk level criteria: 1) at least one confirmed *R*. *rickettsii*-positive tick; 2) a prevalence of RMSF antibodies in dogs > 30%; and 3) a confirmed human case of RMSF within 12 months preceding the study.

### Patterns of movement

**Fig 3:**
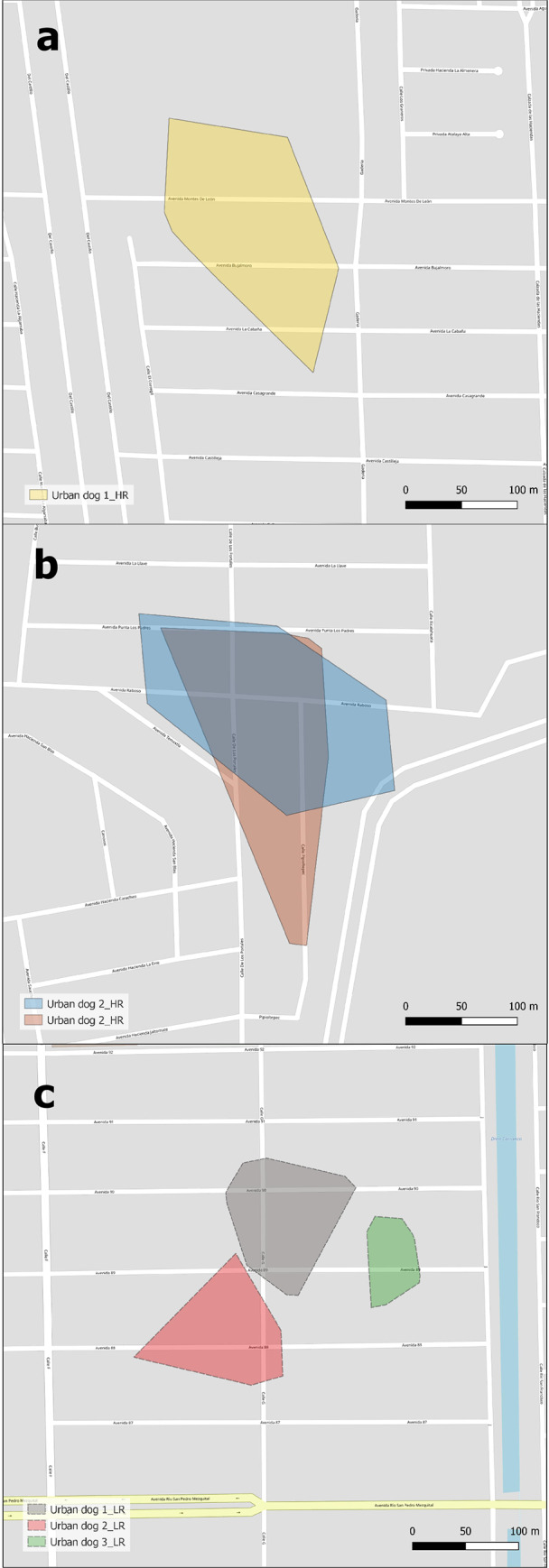
Occupied area of three dogs from a RMSF high-risk neighborhood (a, b), and three dogs from a low-risk neighborhood (c) in Mexicali City. Reprinted from the OpenStreetMap vector basemap hosted by Environmental Systems Research Institute (Esri) and provided under a CC BY 4.0 license, (Map data © OpenStreetMap contributors, Map layer by Esri 2019).

**Fig 4:**
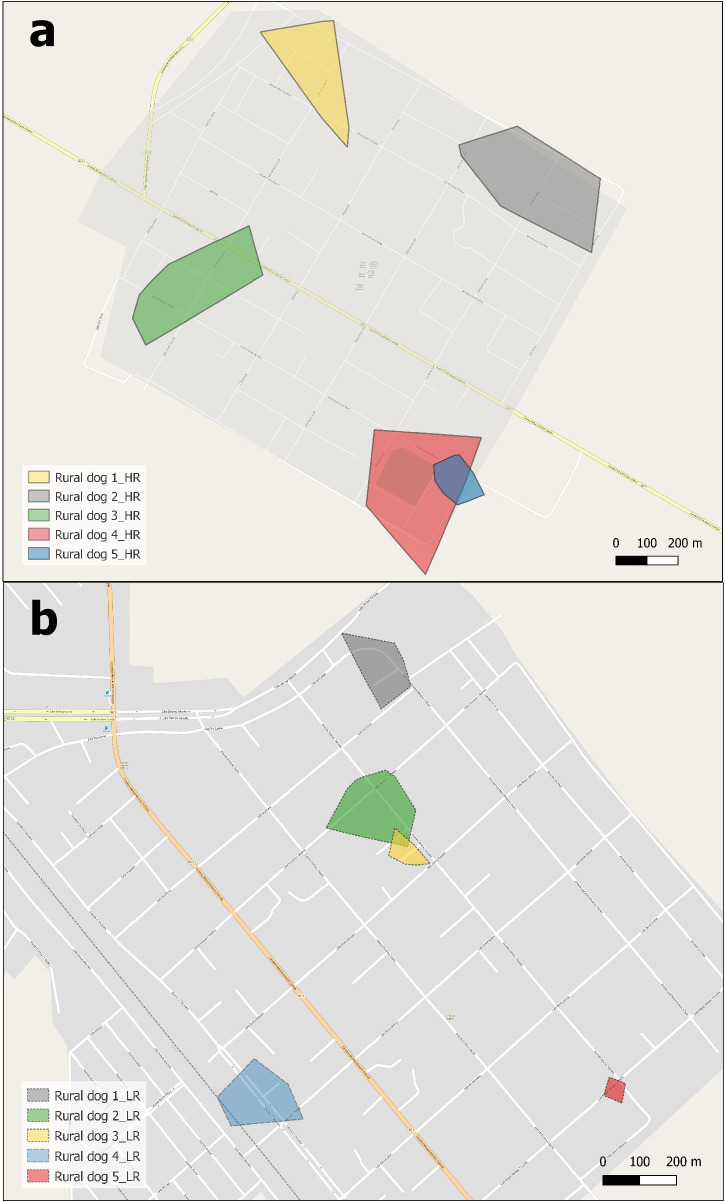
Occupied area of five dogs from RMSF high-risk rural villages (a) and five dogs from low-risk rural villages (b) in Mexicali Valley, Mexico. Reprinted from the OpenStreetMap vector basemap hosted by Environmental Systems Research Institute (Esri) and provided under a CC BY 4.0 license, (Map data © OpenStreetMap contributors, Map layer by Esri 2019).

**Fig 5:**
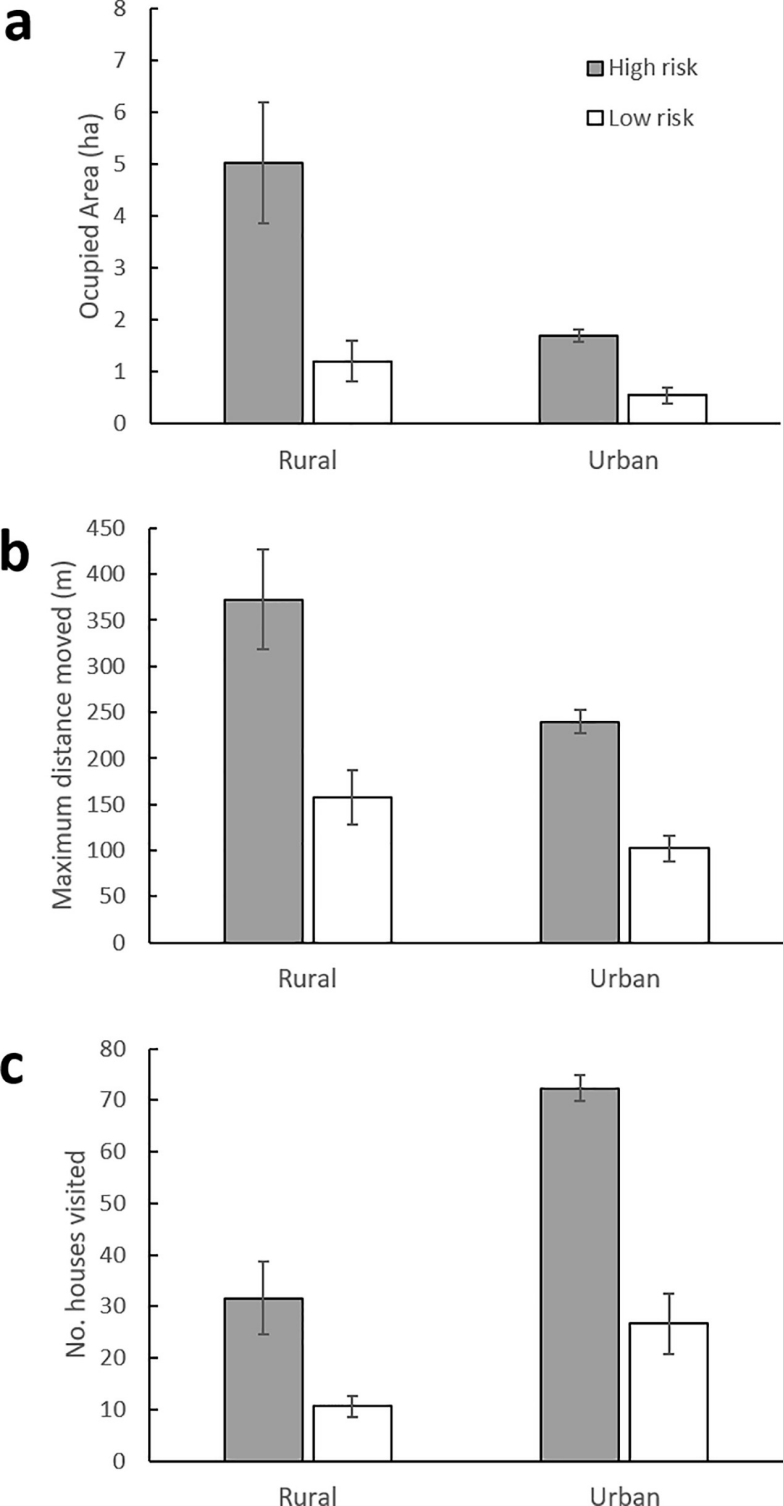
Mean (± SE) occupied area (a), maximum distance moved (b), and number of houses visited (c) by collared dogs in rural and urban areas with two different RMSF risk levels in and near Mexicali, Mexico.

We recorded 4,341 location points, including 3,069 location points from 10 rural dogs and 1,272 location points from 6 urban dogs to summarize movement patterns (Figs 3 and 4). Two-way ANOVA results indicate that mean AO, MDM and NHV differed significantly between risk level and landscape types. The mean AO of dogs from RMSF high-risk areas was significantly higher than for dogs in low-risk areas. Similarly, mean MDM was higher for high-risk vs. low-risk dogs, as were mean number of houses within areas. There were significant differences among AO, MDM, and NHV between rural vs urban areas (Table 5; Fig 5).

**Table 5:** Summary and ANOVA test statistics for the outcomes of area occupied as determined by minimum complex polygon (AO), maximum distance moved (MDM) and total number of houses visited (NHV) among collared dogs from rural and urban areas with two levels of RMSF risk in and near Mexicali, Mexico.

Source of Variation	Level^1^	Mean ± SE	d.f.	F	P
**Area (AO: hectare)**					
Risk level			1	5.81	**0.033**
	High	3.8 ± 0.9			
	Low	0.9 ± 0.3			
Landscape			1	12.45	**0.004**
	Rural	3.1 ± 0.9			
	Urban	1.1 ± 0.3			
Risk*Landscape			1	2.60	0.133
Residuals			12		
**Distance (MDM: meter)**					
Risk level			1	5.08	**0.044**
	High	322.8 ± 40.8			
	Low	136.8 ± 20.8			
Landscape			1	21.13	**0.000**
	Rural	265.1 ± 46.2			
	Urban	170.9 ± 31.9			
Risk*Landscape			1	0.87	0.370
Residuals			12		
**Houses (NH)**					
Risk level			1	27.75	**0.000**
	High	46.9 ± 8.6			
	Low	16.6 ± 3.7			
Landscape			1	33.59	**0.000**
	Rural	21.1 ± 4.9			
	Urban	49.5 ± 10.6			
Risk*Landscape			1	5.23	**0.041**
Residuals			12		

^1^High-risk level criteria: 1) at least one confirmed *R*. *rickettsii*-positive tick; 2) a prevalence of RMSF antibodies in dogs > 30%; and 3) a confirmed human case of RMSF within 12 months preceding the study; Significant p-values are indicated in bold.

## Discussion

Over the last two decades, eastern Arizona and northwestern Mexico have experienced emergence of RMSF vectored by brown dog ticks [16, 24]. Parasitism by brown dog ticks and transmission risk of *R*. *rickettsii* are associated with an increase of free-roaming dogs and the presence of highly infested dogs in the environment [13, 14]. Here, we provide valuable insights into the demography and behavior patterns of dogs in a RMSF epidemic region at the northwestern Mexico-US border. Both rural and urban areas characterized by *R*. *rickettsii*-infected ticks, high canine seroprevalence, and high-risk of human disease [21] tended to have more puppies and dogs that roamed much more widely, but not absolutely higher dog density or numbers of free roaming dogs.

Our results of higher fecundity and litter numbers in high-risk areas suggest that lack of herd immunity due to high puppy recruitment can increase vulnerability in the dog and human populations. Although transovarial and transstadial transmission of *R*. *rickettsii* in brown dog ticks can maintain the infection within a population [20, 27], prevalence in ticks is often below 3% [21, 24], indicating that infected dogs may be important in RMSF epidemics. Once a susceptible dog is infected, it may experience high bacteremia and can serve as a source of rickettsial infection to brown dog ticks, with dogs thus serving as amplifying hosts [19]. Dogs affected by RMSF can develop a similar illness as humans and may die as a result of illness [28]. Dogs recovering from spotted fever rickettsioses are reported to have antibodies that persist at least 6–12 months which, together with cell-mediated immunity, limit or protect against further clinical disease [29, 30]. This suggests a link between the high risk for RMSF in Mexicali and the high proportion of immunologically naïve puppies and increase in the proportion of susceptible dogs.

Reproduction performance of wild and domestic canids can be affected by multiple variables such as body size, age, dog breed, population density and structure, feeding habits, habitat variance, and climate conditions [31–33]. The higher fecundity in high-risk areas could be explained by the population structure of dogs, with a lower proportion of older dogs in these areas. A previous study of dogs found a positive relationship between decline in whelping rate and advancing age [33]. Availability of resources and to what degree humans accept dog presence can also affect reproduction performance in dog populations. Food stress can reduce ovulation rate and lactation, which in turn has consequences on litter size at birth and weaning, respectively [31]. In addition, socioeconomic and cultural factors affect the food availability of, as well as, the dependence of the dog population on environmental resources such as garbage dumps and waste food [34–36]. Nevertheless, although free-roaming dogs were frequently observed scavenging in waste in both rural and urban areas in Mexicali (Lopez-Perez, unpub. data), further investigations are required to elucidate whether or not feeding patterns and quality of food are affecting the reproduction performance of dogs in this site.

The GPS logger provided unique insights on the patterns of domestic dog mobility and their relationship with transmission of RMSF. The marked increase of space use by free-roaming dogs in high-risk areas, as well as the fact that these dogs visited twice as many houses as dogs from low-risk areas, suggest that unrestrained dogs may play an important role in spreading ticks into homes and yards. This is consistent with earlier findings [13] that unrestrained dogs tended to have more ticks than restrained dogs, suggesting that they are more likely to travel to untreated areas such as outlying non peri-domestic sites and acquire ticks.

Movement patterns of free-roaming dogs are determined by several ecological, behavioral, environmental, and socio-economic factors occurring at multiple spatial and temporal scales, including animal fitness, scavenging for food, population density, social interactions, sex and reproductive status, dog ownership practices, human interactions, and landscape structure [37–42]. Two likely explanations for the increased mobility of free-roaming male dogs in high-risk areas of Mexicali could be that, due to food sources, they were foraging farther from their households [37, 39] and that they were seeking intact bitches in estrous [38, 42], given our finding of high fecundity, as well as the slightly greater number of male dogs compared with females.

Our results raise the valid question whether age and sex of dogs affect the dog movement pattern in high and low-risk RMSF areas. Our study featured a relatively small number of collared dogs, and all initial dogs enrolled by chance were male and of similar age; thus our analysis was constrained only to male dogs. Thus we could not test for differences between sex and age. Male dogs often move greater distances and have larger home ranges than females [42, 43] (although these differences are not always detected in the literature [44] and may differ depending on whether dogs are neutered or intact [42]), suggesting that they could spread more ticks than females. Previous studies did not find significant differences in movement patterns among dogs of different ages [42, 45].

Although our findings suggest that dog reproductive performance and turnover are important factors affecting the transmission of RMSF in Mexicali, it is probable that the epidemiological dynamics of the diseases are also influenced by other indirect and direct, multifactorial factors including socioeconomic, biological, and environmental factors at different temporal and spatial scales [13, 18, 21, 46]. For instance, although this study focused on owned free-roaming dogs, dogs of unknown ownership were often observed by study participants, and were significantly more common in rural high- compared with low-risk rural areas (but not in urban areas). Further studies could clarify whether free-roaming dogs are “stray” or owned, because stray and owned free-roaming dogs could differ in their movement patterns and probability of spreading ticks and RMSF, and management of these different canine subsets would differentially involve dog owners or public health and animal control authorities.

Although study limitations, such as the relatively small sample size and the fact that site risk levels were characterized based on a previous study but not long-term epidemiological surveillance, this study represents important data and should be used to motivate further evaluation. Moreover we provide important insight into how dog demography, ownership, and movement patterns might impact the ecology of RMSF in northwestern Mexico. In addition to previous approaches for RMSF control and prevention such as treatment of houses with pesticides, use of a long-acting tick collar [13, 47, 48], our data indicate that policies encouraging changes the population structure of dog by decreasing the proportion of puppies such as through spay-neuter campaigns could enhance herd immunity if the adult dogs have been exposed and developed immunity. Fewer intact bitches could reduce attraction to stray males, and this benefit could further be enhanced by reducing the number of free-roaming dogs. In addition, our results point out that policies encouraging dog restraint to limit the free trafficking of dogs and ticks across communities may have implications as means to limit RMSF risk, rather than the restriction of the number of owned dogs. Finally, use of a vaccine in dogs, such as the recently described whole-cell inactivated antigen vaccine [49], could also reduce risk to humans and dogs by boosting canine herd immunity.

This study emphasizes the importance of a One Health approach and a long term monitoring epidemiological survey to better understand the transmission mechanisms of *R*. *rickettsii* and pathogen-vector-hosts interactions. Better understanding can improve control and prevention of the spread of rickettsioses and other diseases for people and dogs.

## Supporting information

S1 Table Household questionnaires deployed. (DOCX)
