# Supplementary material for: An exploratory analysis of demography and movement patterns of dogs: New insights in the ecology of endemic Rocky Mountain-Spotted Fever in Mexicali, Mexico
Source: PLoS One. 2020 May 21;15(5):e0233567. doi: 10.1371/journal.pone.0233567 (PMC7241830; doi:10.1371/journal.pone.0233567)
Supplement: S1 Table — (DOCX) [file pone.0233567.s001.docx]

No. Questionnaire _____

**HOUSEHOLD QUESTIONNAIRE**

Interviewer: _____________________________________ Date: ________________

Address: ___________________ GPS Location: _________________ __________________

Name of the owner: _______________________________________________________

1. **QUESTIONS RELATED TO THE DOG**
2. How many dogs live here? _____________________________
3. How many puppies? (less 3 months) _______________
4. Dog details

| Name | Sex (M/F) | Age* | Bred (Y/N/U) | Origin^1^ | Function^2^ | Free-ranging (Y/N)^3^ | Sterilized (Y/N)^3^ |
| --- | --- | --- | --- | --- | --- | --- | --- |
|  |  |  |  |  |  |  |  |
|  |  |  |  |  |  |  |  |
|  |  |  |  |  |  |  |  |
|  |  |  |  |  |  |  |  |
|  |  |  |  |  |  |  |  |

^*^If age is unknown, for how long have they owned the dog?

^1^ A: acquired from neighbor or family; B: buy; F: found or adopted; BH: born in the house

^2^ G: guardian; S: shepherd; P: pet; H: hunting

^3^ Y: Yes; N: No

1. If your dog is female answer the questions below (Fertility)

| Name | No. litters | Litter in last 12 months? (Y/N) | If yes, month? | Puppies of last 12 months | |
| --- | --- | --- | --- | --- | --- |
|  |  |  |  | Size of litter | How many remain? |
|  |  |  |  |  |  |
|  |  |  |  |  |  |
|  |  |  |  |  |  |

1. Have you seen free-roaming unknown ownership dogs? Where?
2. YES b) NO

No. de encuesta _____

**Encuesta para dueño de perros**

Entrevistador: _____________________________________ Fecha: ________________

Dirección: ___________________ Localidad GPS: _________________ __________________

Nombre del dueño: _______________________________________________________

1. **Preguntas relacionadas a los perros**
2. ¿Cuántos perros viven en su casa? _____________________________
3. ¿Cuántos cachorros tienen? (menos de 3 meses) _______________
4. Detalles de los perros:

| Nombre | Sexo (M/H) | Edad* | ¿Se ha apareado? (S/N) | Origen^1^ | Función^2^ | Libre Acceso  (S/N)^3^ | Esterilizado (S/N)^3^ |
| --- | --- | --- | --- | --- | --- | --- | --- |
|  |  |  |  |  |  |  |  |
|  |  |  |  |  |  |  |  |
|  |  |  |  |  |  |  |  |
|  |  |  |  |  |  |  |  |
|  |  |  |  |  |  |  |  |

^*^Si la edad no es conocida, ¿Cuánto tiempo ha tenido al perro?

^1^ A: adquirido de un vecino o familiar; C: comprado; E: encontrado o adoptado; NC: Nacido en casa

^2^ G: Guardián; P: Pastor; M: Mascota; C: Cacería

^3^ S: SI; N: No

1. Si tienen hembras contestar las preguntas siguientes (Fertilidad)

| Nombre | No. camada | ¿Camada en los últimos 12 meses? (S/N) | Si, si, ¿en qué mes? | Cachorros en los últimos 12 meses | |
| --- | --- | --- | --- | --- | --- |
|  |  |  |  | Tamaño de camada | ¿Cuántos permanecen en casa? |
|  |  |  |  |  |  |
|  |  |  |  |  |  |
|  |  |  |  |  |  |

1. ¿Has visto perros sin restricción sin dueño?
2. SI b) NO
